# Dasatinib blocks transcriptional and promigratory responses to transforming growth factor-beta in pancreatic adenocarcinoma cells through inhibition of Smad signalling: implications for in vivo mode of action

**DOI:** 10.1186/s12943-015-0468-0

**Published:** 2015-11-21

**Authors:** Tobias Bartscht, Benjamin Rosien, Dirk Rades, Roland Kaufmann, Harald Biersack, Hendrik Lehnert, Frank Gieseler, Hendrik Ungefroren

**Affiliations:** First Department of Medicine, UKSH, Campus Lübeck, 23538 Lübeck, Germany; Department of Radiation Oncology, UKSH, Campus Lübeck, D-23538 Lübeck, Germany; Department of General, Visceral and Vascular Surgery, Jena University Hospital, D-07747 Jena, Germany

**Keywords:** Dasatinib, TGF-β, Smad, PDAC, Cell migration, Invasion, Activin receptor-like kinase 5

## Abstract

**Background:**

We have previously shown in pancreatic ductal adenocarcinoma (PDAC) cells that the SRC inhibitors PP2 and PP1 effectively inhibited TGF-β1-mediated cellular responses by blocking the kinase function of the TGF-β type I receptor ALK5 rather than SRC. Here, we investigated the ability of the clinically utilised SRC/ABL inhibitor dasatinib to mimic the PP2/PP1 effect.

**Methods:**

The effect of dasatinib on TGF-β1-dependent Smad2/3 phosphorylation, general transcriptional activity, gene expression, cell motility, and the generation of tumour stem cells was measured in Panc-1 and Colo-357 cells using immunoblotting, reporter gene assays, RT-PCR, impedance-based real-time measurement of cell migration, and colony formation assays, respectively.

**Results:**

In both PDAC cell lines, dasatinib effectively blocked TGF-β1-induced Smad phosphorylation, activity of 3TPlux and pCAGA_(12)_-luc reporter genes, cell migration, and expression of individual TGF-β1 target genes associated with epithelial-mesenchymal transition and invasion. Moreover, dasatinib strongly interfered with the TGF-β1-induced generation of tumour stem cells as demonstrated by gene expression analysis and single cell colony formation. Dasatinib also inhibited the high constitutive migratory activity conferred on Panc-1 cells by ectopic expression of kinase-active ALK5.

**Conclusions:**

Our data suggest that the clinical efficiency of dasatinib may in part be due to cross-inhibition of tumour-promoting TGF-β signalling. Dasatinib may be useful as a dual TGF-β/SRC inhibitor in experimental and clinical therapeutics to prevent metastatic spread in late-stage PDAC and other tumours.

**Electronic supplementary material:**

The online version of this article (doi:10.1186/s12943-015-0468-0) contains supplementary material, which is available to authorized users.

## Background

Despite significant progress in the biology of pancreatic ductal adenocarcinoma (PDAC), treatment options for affected patients are still very limited and far from being curative with the exception of surgery (R0 resection). Chemotherapy is complicated by the desmoplastic nature of the complex mircoenvironmental architecture of the tumour tissue which is known to favour metastasis and chemoresistance of tumour cells.

Due to its overexpression in PDAC and its various functions in driving tumour suppression, the non-receptor tyrosine kinase SRC represents a promising biological target in experimental and clinical approaches to treat PDAC [1–3]. Dasatinib (N-(2-chloro-6-methyl- phenyl)-2-(6-(4-(2-hydroxyethyl)- piperazin-1-yl)-2-methylpyrimidin-4- ylamino)thiazole-5-carboxamide; BMS-354825, Sprycel), a tyrosine kinase inhibitor originally developed against BCR-ABL and SRC [4] and currently used in the treatment of CML [5] and Philadelphia chromosome-positive acute lymphoblastic leukemia (reviewed in [6]), has also shown promise in the treatment of various epithelial tumours [6] including pancreatic cancer. Dasatinib-mediated inhibition of SRC slowed tumour progression and metastasis of human PDAC cells in an orthotopic mouse model [7], and stimulated migration, invasion, and apoptosis in human [8] and murine [9] PDAC cells. Moreover, dasatinib inhibited metastasis in a mouse model of PDAC but failed to suppress primary tumour growth and prolong survival [7]. Along the same lines, a phase II study found that dasatinib as a single-agent did not have clinical activity as first-line therapy in patients with metastatic PDAC [10].

Several preclinical studies have shown that dasatinib can potentiate the antitumoural action of various other anti-cancer drugs (reviewed in [11]. For instance, dasatinib synergized with gemcitabine to induce anti-proliferation and apoptosis in the pancreatic cancer cell line MIA PaCa-2 by decreasing the levels of ALDH1A1, a marker of tumour-initiating/cancer stem cells [12]. Interestingly, 2/8 patients with pancreatic cancer (both gemcitabine-refractory) who received both gemcitabine and dasatinib showed a partial response (stable disease ≥6 months) [13]. When dasatinib was combined with gemcitabine *and* erlotinib (an epidermal growth factor-receptor (EGF-R) inhibitor), it inhibited the growth of xenografts of both sensitive and resistant PDAC cells in vivo without increasing toxicity [14]. More recently, concomitant targeting of SRC, EGF-R, and transforming growth factor (TGF)-β has been suggested as a novel therapeutic approach in pancreatic cancer [15].

Although originally developed as an inhibitor of BCR-ABL and SRC [16], dasatinib, in drug affinity chromatography experiments was shown to interact with over 40 kinases, including SRC family kinases (SFKs), receptor tyrosine kinases, serine/threonine kinases (STK), MAP kinases, and EphA2 [17]. One of the STKs identified with this approach was the type I receptor for TGF-β (TβRI, also termed activin receptor-like kinase 5, ALK5) [18]. TGF-β1 is a pleiotropic growth factor that controls several aspects of tumour cell behavior such as proliferation, angiogenesis, desmoplasia, cell migration/invasion, and metastasis. It has a central role in the initiation and progression of PDAC [19] which is evident from the observation that its aberrant expression in advanced tumour stages is associated with decreased survival in PDAC patients [20], and that the TGF-β1 signalling pathway is among the 12 core pathways that are genetically altered in 100 % of PDAC tumours [21]. Besides ALK5, TGF-β1 requires a second membrane-bound STK receptor, designated type II (TβRII), for signal transmission into cells. Upon phosphorylation by TβRII, ALK5 initiates canonical Smad as well as non-Smad signalling pathways [22] that together mediate the promigratory and proinvasive effects of TGF-β. For PDAC, this is evident from the Panc-1 orthotopic mouse model in which ectopic expression of kinase-active ALK5 (ALK5^T204D^) strongly enhanced metastasis [23] while pharmacologic inhibition of endogenous ALK5 suppressed it [24]. Targeting ALK5 in vivo is therefore a feasible approach to the treatment of PDAC and other carcinomas.

Like SRC, TGF-β/ALK5 signalling is currently targeted in the experimental and clinical treatment of various tumours. Given i) the interaction of dasatinib with ALK5 [18, 25], ii) the structural similarity of dasatinib with the experimental SRC inhibitors PP2 and PP1, and iii) the ability of PP2 and PP1 to effectively inhibit the ALK5 kinase activity as well as TGF-β1-induced prooncogenic responses [26, 27], we hypothesized that dasatinib should be able to block TGF-β1 signalling towards migratory, invasive and prometastatic outcomes. That dasatinib may possess potential efficacy against profibrotic TGF-β signalling in vivo was suggested by preclinical studies, in which dasatinib treatment of scleroderma and normal fibroblasts led to decreased production of extracellular matrix proteins [28]. In light of the clinical use and efficacy of dasatinib, it is mandatory to understand its molecular mode of action in vivo including possible side-effects, regardless of whether they are adverse or beneficial for the patients.

To investigate the effect of dasatinib on TGF-β/ALK5 signalling in PDAC, we employed two TGF-β sensitive cell lines (Panc-1, Colo-357) that have been used in orthotopic mouse models of PDAC for evaluation of TGF-β antitumour activity in vivo [23, 29]. Using impedance-based real-time measurement of cell migration, we show here that dasatinib strongly and dose-dependently inhibited TGF-β1-induced migratory responses *in vitro*. As a result of dasatinib inhibition, activation of Smad2/3, upregulation of TGF-β transcriptional reporter genes as well as EMT/migration/invasion-associated gene expression in PDAC-derived cell lines was compromised. These results have implications for the use of dasatinib in experimental therapeutics, providing a molecular explanation for the reduction in the cells’ migratory response and an in vivo correlate for its anti-metastatic action. Furthermore, our data suggest that this agent based on its dual effect on protumourigenic TGF-β and SRC signalling pathways may be useful in the treatment of late stage metastatic disease in PDAC.

## Results

### Dasatinib blocks TGF-β1-induced Smad2/3 activation in PDAC cells

**Fig. 1:**
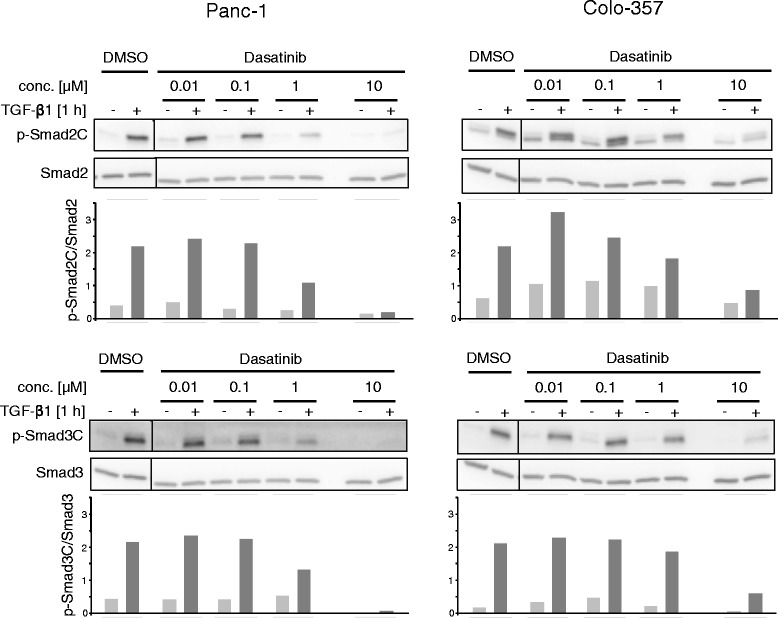
Immunoblot analysis of TGF-β1-induced R-Smad activation in Panc-1 and Colo-357 cells treated with dasatinib. Panc-1 and Colo-357 cells were stimulated with 5 ng/ml TGF-β1 for 1 h in the presence of vehicle (DMSO, 0.1 % *v/v*) or the indicated concentrations of dasatinib, and subsequently evaluated by immunoblotting for C-terminal phosphorylation of Smad2 (p-Smad2C, upper two panels) and Smad3 (p-Smad3C, lower two panels). Equal loading of total protein was verified with antibodies to Smad2 and Smad3, respectively. Below the immunoblots is a graphic representation of the results from densitometric analysis of p-Smad band intensities after normalization with those for the respective total Smad proteins

In order to test whether dasatinib can inhibit ALK5 function, we measured its effect on TGF-β1-induced C-terminal phosphorylation of Smad2 and Smad3 (p-Smad2C/3C), reflecting their state of activation. To this end, dasatinib dose-dependently inhibited TGF-β1-induced p-Smad2C and p-Smad3C in both Panc-1 and Colo-357 cells with the greatest effects seen at concentrations >0.1 μM (Fig. 1). A comparison of signal strengths between Panc-1 and Colo-357 cells after quantification by densitometry revealed that the dasatinib effect on both p-Smads was more pronounced in Panc-1 cells (Fig. 1).

### Dasatinib blocks TGF-β1-induced reporter gene activity

**Fig. 2:**
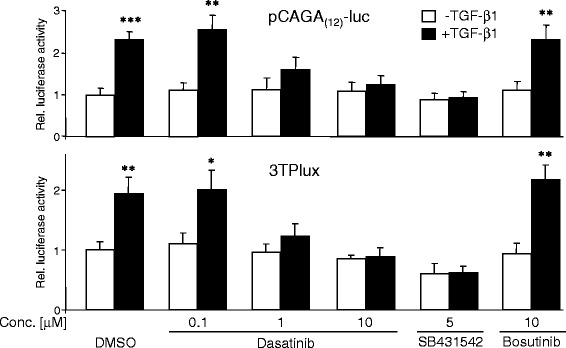
Treatment of Panc-1 cells with dasatinib decreases the sensitivity of TGF-β/Smad-responsive reporters to TGF-β1 stimulation. Panc-1 cells were transfected on day 1 with either pCAGA_(12)_-luc (upper graph) or 3TPlux (lower graph), and the *Renilla* luciferase-encoding vector pRL-TK-luc using LipofectAmine 2000. Twenty-four h after the start of transfection, cells were stimulated with TGF-β1 for another 24-h period in the presence of 0.1 % DMSO or the indicated concentrations of dasatinib, SB431542, or bosutinib, followed by dual luciferase measurements. Data represent firefly luciferase values normalised with those for *Renilla* luciferase (displayed as relative (rel.) luciferase activities with those for DMSO/non-TGF-β1-treated cells set arbitrarily at 1) and are the mean ± SD from three independent assays. Asterisks indicate significance of TGF-β1-treated cells *vs.* the respective untreated control (*n* = 3)

The above data showed that TGF-β1-induced Smad2 and Smad3 activation was sensitive to dasatinib inhibition. Hence, we wondered whether this effect of dasatinib on the central mediators of TGF-β signalling would have consequences for Smad-mediated transcriptional activation of TGF-β1 target genes. To this end, cells were transiently transfected with the TGF-β reporter genes pCAGA_(12)_-luc or 3TPlux. While pCAGA_(12)_-luc is Smad-specific and responds to binding of Smad3/4 rather than Smad2/4 to the Smad binding elements (SBEs) in this plasmid [30], the 3TPlux plasmid contains promoter sequences from the plasminogen activator-inhibitor-1 gene and is responsive to both Smad and non-Smad pathways [31]. Treatment of Panc-1 cells with a combination of TGF-β1 and dasatinib strongly suppressed the response of both pCAGA_(12)_-luc and 3TPlux to TGF-β1 induction in a dose-dependent manner (Fig. 2), and at a concentration of 10 μM was equally effective in suppressing reporter gene activity as the established ALK5 inhibitor SB431542 at 5 μM (Fig. 2). In contrast, the SRC inhibitor bosutinib (SKI-606, IC_50_ = 1.2 nM) was unable to interfere with TGF-β transcriptional activity. Thus, dasatinib appears to be a powerful inhibitor of transcriptional responses to TGF-β in PDAC-derived cell lines.

### Dasatinib blocks TGF-β1-induced expression of genes associated with EMT, migration/invasion and stemness

**Fig. 3:**
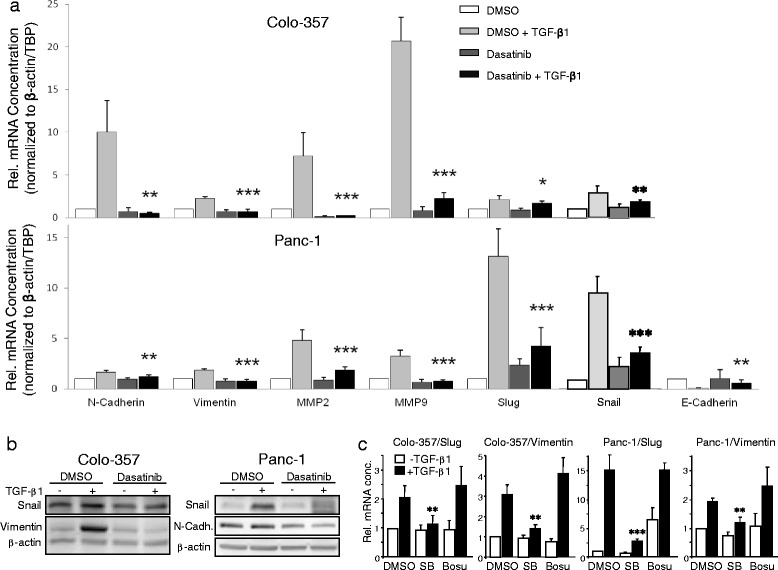
Dasatinib blocks TGF-β1-induced expression of genes involved in EMT and cell migration/invasion. **a** Colo-357 (upper graph) and Panc-1 cells (lower graph) cultured for 24 h in the absence or presence of TGF-β1 (5 ng/ml) and either DMSO or dasatinib (10 μM) were subjected to RNA isolation and qPCR-based determination of the indicated genes. Data are plotted relative to untreated DMSO-treated control cells (set arbitrarily at 1) and represent the mean ± SD from three independent experiments after normalisation to β-actin and TBP. *Asterisks* indicate a significant difference between dasatinib + TGF-β1-treated cells and DMSO + TGF-β1-treated cells (*n* = 3). **b** Colo-357 and Panc-1 cells cultured for 48 h in the absence or presence of TGF-β1 (5 ng/ml) and either DMSO or dasatinib (10 μM) were subjected to immunoblot analyses of the indicated proteins and β-actin as a loading control. One representative blot is shown for each cell line. **c** Colo-357 and Panc-1 cells cultured for 24 h in the absence or presence of TGF-β1 (5 ng/ml) and either DMSO, SB431542 (SB, 5 μM) bosutinib (Bosu, 10 μM) were subjected to qPCR-based determination of Slug and vimentin. Data represent the mean ± SD from three independent experiments. *Asterisks* indicate a significant difference between SB + TGF-β1-treated cells and DMSO + TGF-β1-treated control cells (*n* = 3)

Next, we asked whether dasatinib would also affect TGF-β1-induced upregulation of genes involved in EMT, cell migration, and invasion. Dasatinib at 10 μM effectively prevented the TGF-β1-induced downregulation of E-cadherin in Panc-1 cells and dramatically inhibited TGF-β1 induction of N-cadherin, vimentin, MMP2, MMP9, Slug and Snail in Colo-357 and Panc-1 cells as demonstrated by quantitative RT-PCR (qPCR, Fig. 3a) and immunoblotting (Fig. 3b). Although TGF-β induction of Snail protein in Colo-357 cells and N-cadherin protein in Panc-1 cells was only moderate, levels were clearly reduced upon dasatinib treatment (Fig. 3b). To show that the dasatinib-induced changes were caused by inhibition of ALK5 rather than SRC, we treated Colo-357 and Panc-1 cells, as above in the reporter gene assays (Fig. 2), with either SB431542 or bosutinib and repeated the qPCR analysis for two central regulators of TGF-β-induced EMT, Slug and vimentin (Fig. 3c). While SB431542 effectively abolished the TGF-β effect on Slug and vimentin mRNA levels in both cell lines, bosutinib lacked statistically significant effects (Fig. 3c).

**Fig. 4:**
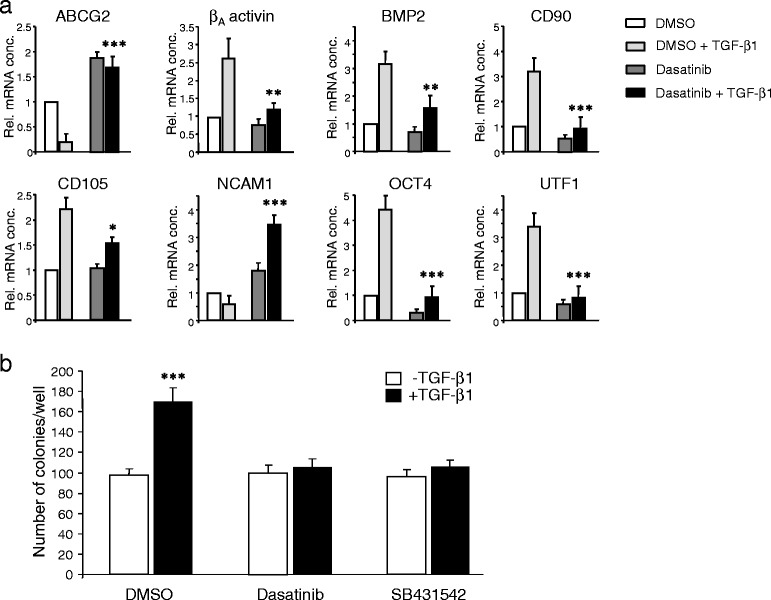
Dasatinib blocks the TGF-β1-induced generation of cells with stem cell character. **a** Panc-1 cells were treated or not for 14 d with TGF-β1 (5 ng/ml) and either DMSO or dasatinib (10 μM) followed by qPCR-based determination of the indicated stem cell-associated genes. Data are plotted relative to untreated DMSO-treated control cells and represent the mean ± SD from three independent experiments. *Asterisks* indicate a significant difference between the DMSO + TGF-β1 and the dasatinib + TGF-β1 treated cells (*n* = 3). **b** Panc-1 cells were treated or not for 14 d with TGF-β1 (5 ng/ml) and either DMSO, dasatinib (10 μM), or SB431542 (5 μM) followed by colony formation assay. Data represent the mean ± SD from three independent assays. *Asterisks* indicate a significant difference relative to the non-TGF-β1-treated control cells (*n* = 3)

Since this gene expression profile suggested that dasatinib might be able to interfere with TGF-β1-induced EMT and partial EMT has been shown to be associated with the aquisition of stem cell traits [32], we subjected Panc-1 cells to a long-term treatment with TGF-β1 according to a previously published protocol [32] to study the effect of dasatinib on the induction of stem cell gene expression. To this end, a 14-day exposure to TGF-β1 resulted in the upregulation of several pluripotency-associated genes: The TGF-β ligand superfamily members β_A_ activin and BMP2, the membrane-associated markers CD90, CD105 (also termed endoglin, a TGF-β coreceptor), and the transcriptional regulators OCT4 (the stem cell associated OCT4A isoform) and UTF1 (Fig. 4). Moreover, we observed downregulation of the adhesion molecule NCAM1 (Fig. 4a) and, surprisingly, of the xenobiotic transporter ATP-binding cassette sub-family G member 2 (ABCG2) (Fig. 4). Intriguingly, the TGF-β1 effect on all genes tested was effectively inhibited by concomitant treatment with dasatinib (Fig. 4a). To verify the possibility that dasatinib is able to block the TGF-β/EMT-induced generation of cells with stem cell character, we repeated the 14-day treatment of Panc-1 cells with TGF-β1 in the absence or presence of dasatinib, or SB431542 as control (long-term treatment of these cells with bosutinib was not possible due to its toxicity). Subsequently, we employed the colony formation assay (CFA) to assess the number of cells with stem cell features by virtue of their ability to generate from a single cell a microscopically discernible clone or colony [33, 34]. A quantitative analysis by counting revealed that both dasatinib, and SB431542 as control, strongly decreased the number of colonies/clones that arose from TGF-β1-treated cultures relative to untreated controls (Fig. 4b). Together, our data suggest that dasatinib interferes with TGF-β-induced EMT and stem cell generation and raise the possibility that it can block the generation of tumour-initiating cells in vivo.

### Dasatinib blocks TGF-β1-induced cell migration

**Fig. 5:**
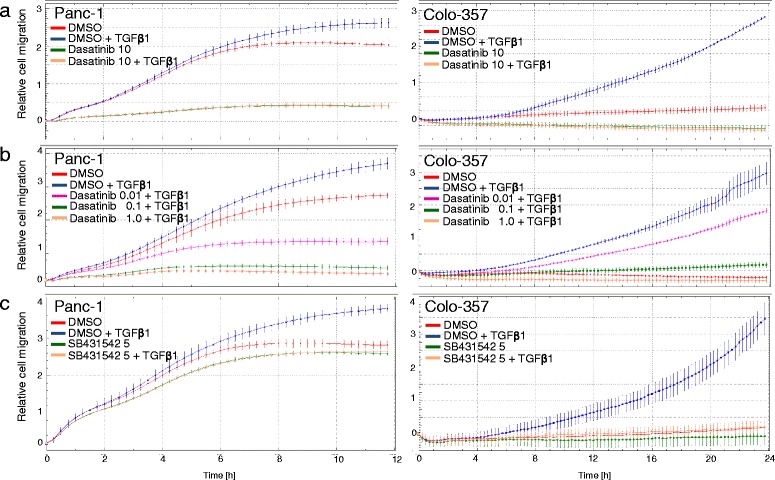
Effect of dasatinib on TGF-β1-induced cell migration in PDAC cell lines. Overnight starved Panc-1 or Colo-357 cells were resuspended in growth medium with 1 % FCS and seeded into the wells of a CIM-Plate 16 (60,000/well) of the RTCA DP instrument. Cells were allowed to migrate in the presence of TGF-β1 (5 ng/ml, added to both the lower and upper compartment of each well) and either vehicle or dasatinib at the indicated concentrations (**a** & **b**) or SB431542 (**c**) as indicated by the *colour code*. Changes in impedance resulting from cells that have migrated to the bottom side of the membranes were recorded every 15 min and monitored for a total of 12 h (Panc-1) and 24 h (Colo-357). Data are from one representative experiment out of three experiments performed in total and are presented as means ± SD of quadruplicate wells. In each graph, significance was calculated for vehicle + TGF-β1 *vs.* dasatinib + TGF-β1 (**a** & **b**) or *vs.* SB431542 + TGF-β1 (**c**). The first time point at which differences become significant are for Panc-1 cells: 10 μM: 0:30 (**a**, *left-hand graph*); 1 μM: 0:15; 0.1 μM: 0:15; 0.01 μM: 0:30 (**b**, *left-hand graph*); 5 μM: 0:30 (**c**, *left-hand graph*); and for Colo-357 cells: 10 μM: 0:47 (**a**, *right-hand graph*); 1 μM: 1:04; 0.1 μM: 3:50; 0.01 μM: 4:35 (**b**, *right-hand graph*); 5 μM: 0:30 (**c**, *right-hand graph*)

Given the ability of dasatinib to block TGF-β1-induced EMT and cell motility-associated gene expression as well as Smad activation and transcriptional activity in conjunction with the requirement for Smad3 for TGF-β1-dependent migration of PDAC cells [35], we hypothesized that dasatinib also interferes with TGF-β1-induced cell motility in PDAC cells. To this end, dasatinib at the 10 μM concentration abolished both basal and TGF-β1-stimulated cell migration in both Panc-1 and Colo-357 cells (Fig. 5a). To determine whether this antimigratory effect of dasatinib was dose-dependent and to be able to calculate the concentration of half-maximal inhibition, we serially diluted the dasatinib and repeated the migration assays with lower concentrations. Interestingly, a concentration as low as 0.01 μM afforded an approximately 50 % reduction in migration activity in both cell types (Fig. 5b, magenta curves). With respect to inhibition of TGF-β1-stimulated migration, the dasatinib effect was comparable to that of SB431542 affording complete inhibition (Fig. 5c), however, dasatinib unlike SB431542 also suppressed the increase in basal migratory activity which in contrast to Colo-357 cells was quite high in Panc-1 cells (Fig. 5c). Interestingly, we have shown earlier that the high basal migration (which precedes TGF-β1-stimulated migration) was SRC-dependent [36], suggesting that in Panc-1 cells dasatinib acts as a dual inhibitor of ALK5 and SRC-mediated migration. Together, these results clearly show that dasatinib, in a very sensitive and effective fashion, blocked TGF-β1-induced migration/invasion in PDAC cells *in vitro*. This data together with those presented in Figs. 1, 2, and 3 strongly support our contention that dasatinib represents a powerful inhibitor of TGF-β1-induced cell motility by inhibiting ALK5 rather than SRC or other SFKs.

### Dasatinib inhibits the high constitutive migratory activity of Panc-1 Cells expressing kinase-active ALK5

**Fig. 6:**
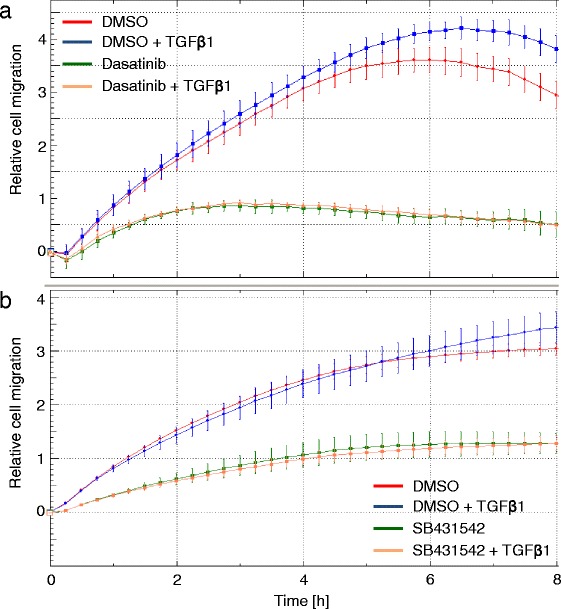
Dasatinib inhibits cell migration induced by kinase-active ALK5. Cells (60,000/well) from a previously characterised clone of Panc-1-ALK5^T204D^ cells were seeded into a CIM-Plate 16 for measurement of cell migration. Cells were allowed to migrate in the absence or presence of exogenous TGF-β1 (5 ng/ml) and either DMSO (0.1 %) or dasatinib (10 μM) (**a**) or SB431542 (5 μM) (**b**). Data shown are given as mean ± SD of quadruplicate wells and were derived from one representative experiment out of three experiments performed in total. Significant differences in the cell index values between vehicle treated (*red curve*) and dasatinib-treated (*green curve*) Panc-1-ALK5^TD^ cells, and between vehicle + TGF-β1 treated (*blue curve*) dasatinib + TGF-β1-treated (*ochre curve*) were first observed at the 1:00 h and all later time points

As shown previously, the experimental SRC inhibitors PP2 and PP1 strongly interfered with the high constitutive migratory activity conferred on these cells by ectopic expression of ALK5^T204D^, a kinase-active version of ALK5 that harbors an activating point mutation in its glycine- and serine-rich (GS) domain [37] and does not rely on ligand binding, complex formation and phosphorylation by the TβRII kinase in order to signal. To test whether dasatinib was capable of mimicking the PP1/PP2 effect, we subjected Panc-1-ALK5^T204D^ cells (one of several individual and previously characterised clones [23]) to the RTCA migration assay. Notably, both dasatinib (Fig. 6a) and SB431542 (Fig. 6b) strongly reduced both migration directed by the exogenously expressed ALK5^T204D^ receptors and the extra portion induced by stimulation of endogenous (non-mutant) receptors in response to their activation by recombinant TGF-β1 ligand. These data are consistent with the results from dasatinib inhibition of ligand-induced cell migration in wild-type cells (see Fig. 5) and confirm that dasatinib targets the ALK5 kinase function or events downstream thereof for inhibition.

## Discussion

The tyrosine kinase inhibitor dasatinib at low concentrations (IC <1.0 nM) potently inhibits ABL and SFKs. At higher concentrations it also inhibits other TKs and STKs such as ALK5, p38 MAPK, AKT, and FAK, all of which have been implicated in TGF-β signalling either in the canonical branch (ALK5), as downstream mediators of Smads (AKT, FAK, p38 MAPK) or in the non-canonical branch (p38 MAPK, FAK) [22]. ALK5 was originally identified by drug affinity chromatography to interact directly with dasatinib [18]. Later, this observation was confirmed in docking studies using the previously reported crystal structure of the ALK5 cytoplasmic domain. Here, the binding of dasatinib to ALK5 presented with the highest score when compared to the structurally related bosutinib, the commercially available ALK5 inhibitor LY-364947, and dorsomorphin [25].

Likewise, we have shown previously that PP2 and PP1, which share structural similarity with dasatinib, effectively attenuated various oncogenic responses to TGF-β1 in malignant PDAC cell lines and strongly inhibited ALK5 kinase activity in *in vitro* kinase assays [26]. In the present study, we have tested in the Panc-1 and Colo-357 cell lines the prediction that dasatinib can mimic the action of PP2 and PP1 on TGF-β signalling. We found that dasatinib effectively blocked TGF-β1-dependent reporter gene activity, Smad2/3 activation and cell migration. The inhibitory effect of dasatinib (IC_50_ = 0.8 nM for SRC in cell-free assays) on ligand-induced reporter gene activity was not dependent on SRC since it was not mimicked by another SRC inhibitor, bosutinib (IC_50_ = 1.2 nM, [38]). Moreover, dasatinib strongly inhibited migration driven by the ALK5^T204D^ mutant (Fig. 6). The inhibitory effect of dasatinib on TGF-β1/ALK5-mediated cell motility corresponded well with the potency of this agent to inhibit various TGF-β1-regulated marker genes involved in EMT, migration/invasion, and a cancer stem cell phenotype. Consistent with this, qPCR assays revealed that dasatinib suppressed TGF-β1 induction of MMP2, MMP9, N-cadherin, vimentin, Snail and Slug in Panc-1 and Colo-357 cells. In Panc-1 cells, dasatinib also attenuated downregulation of the adhesion molecules E-cadherin and NCAM1, together providing a molecular explanation for the anti-migratory/anti-invasive effect of dasatinib. Moreover, dasatinib effectively prevented the TGF-β1-induced upregulation of several stem cell-associated genes, some of which are either members of the TGF-β superfamily of ligands (β_A_ activin, BMP2) or function as a co-receptor for TGF-β (CD105/endoglin) and strongly decreased the number of colony-forming units derived from long-term TGF-β1-treated cells assumed to derive from cancer stem cells. From these data, we conclude that dasatinib targets ALK5 for inhibition of TGF-β-induced cell motility *in vitro* and likely also other EMT-associated changes such as cancer stem cell differentiation.

Gordian and colleagues [25] studied the combined effects of dasatinib and TGF-β on A549 NSCLC cells. When combined with TGF-β1 stimulation, dasatinib induced apoptosis in EGF-R mutant cells along with upregulation of pro-apoptotic BIM protein. Unfortunately, cell motility responses were not analysed in this study. For analysis of Smad2 and Smad3 activation by TGF-β1, these authors used a single concentration of 100 nM dasatinib rather than providing a dose–response curve with higher concentrations. With this dose they observed an increase in the levels of p-Smad3C compared to levels seen with TGF-β1 alone. Interestingly, we also observed in PDAC cells a tendency for an increase or at least a lack of effect in the concentration range of 10–100 nM, which was particularly evident in the Colo-357 cell line (see Fig. 1).

Several drugs have been developed for blocking TGF-β signalling that are in various stages of experimental and clinical evaluation. Strategies for inhibition of TGF-β signalling include blocking i) the binding of TGF-β to its receptors, ii) the ALK5 kinase using small molecules that act as competitive inhibitors for its ATP-binding site (SB431542, SB505124, SD-093, SD-208, LY580276, and Y2109761, a novel TβRI and TβRII dual inhibitor), and iii) Smad intracellular signal transduction via the Smad3 inhibitor SIS3 [39]. However, although many of these drugs show promise in pre-clinical studies, the dual role of TGF-β in tumour progression requires a deeper understanding of the TGF-β signalling crosstalk with other pathways in order to design successful therapeutic approaches and protect the patients from undesired side effects.

Taken together, our results clearly show that dasatinib can block the TGF-β1-dependent cell motility at concentrations as low as 0.01 μM. However, this extremely sensitive and potent effect of dasatinib, particularly in Panc-1 cells (see Fig. 5), may have resulted from combined inhibition of ALK5 *and* SRC since we have previously shown that SRC contributed to TGF-β1-mediated cell migration without affecting TGF-β1/ALK5-induced activation of Smad2 and Smad3. This differential contribution of SRC may explain why dasatinib was able to block cell migration at much lower concentrations than phosphorylation of Smad2C/3C (compare Figs. 1 and 5). The higher efficacy of dasatinib in blocking TGF-β1-induced cell motility may be due to co-inhibition of p38 MAPK, the activation by TGF-β1 of which is SRC-dependent [40] and required for cell migration in Panc-1 cells (H.U., unpublished observation). Experiments are currently underway to solve this issue. That dasatinib likely acted on SRC in the migration assays was evident from its suppressing effect on basal migration activity in Panc-1 cells which was previously shown to be SRC-dependent [36]. Notably, TGF-β stimulation of p38 MAPK activation and cell invasion requires SRC to phosphorylate TβRII [40]. Because this event is upstream of ALK5 activation, SRC is unlikely to be involved in ALK5^T204D^-mediated cell migration consistent with the very similar migration curves of dasatinib and SB431542-treated cells.

In the light of our results, the possibility remains that dasatinib’s therapeutic effects result to a large extent from inhibition of TGF-β signalling rather than signalling by its *bona fide* target SRC. That dasatinib can indeed compromise undesired effects of TGF-β in vivo was suggested by preclinical studies in an *in vitro* model of a fibrosing disorder in which dasatinib treatment of scleroderma and normal fibroblasts led to decreased production of extracellular matrix proteins [28]. The suppressive effect of dasatinib on TGF-β signalling is likely mediated by inhibition of the ALK5 kinase activity and may be exploited therapeutically in more advanced PDAC (when TGF-β1 expression and SRC activity are high) to synergistically reduce invasion, metastasis and eventually cancer stem cell formation. Consistent with this, concomitant targeting of EGF-R, TGF-β and SRC has been suggested as a novel therapeutic approach in pancreatic cancer [15].

## Conclusions

Understanding how anti-cancer drugs act in vivo is of utmost importance for risk and treatment stratification and for developing targeted combination therapies in advanced PDAC. TGF-β1 is a prometastatic ligand in pancreatic cancer which regulates EMT, cell migration/invasion and cancer stem cell formation. Here, we show that the BCR-ABL/SRC inhibitor dasatinib strongly interfered with these tumour-promoting responses, suggesting that the clinical efficiency of dasatinib may be due in part to cross-inhibition of TGF-β/ALK5 signalling. Hence, dasatinib may be useful as a dual TGF-β/SRC inhibitor in experimental and clinical therapeutics to prevent metastatic spread in late-stage PDAC and other tumours.

## Methods

### Reagents

TGF-β1 was obtained from ReliaTech (Wolfenbüttel, Germany). Dasatinib was provided by Bristol-Myers Squibb (UK) and bosutinib (SKI-606) was kindly donated by Pfizer (USA). Stocks of these compounds were prepared in dimethyl sulfoxide (DMSO) which at the concentration used (0.1 %) had no measurable effect on cellular activities (data not shown).

### Cell lines, cell culture and treatment with TGF-β1 and inhibitors

The PDAC cell lines Panc-1 and Colo-357 were maintained in standard culture medium consisting of RPMI 1640 supplemented with 10 % fetal calf serum (FCS), L-glutamine, sodium pyruvate and 50 units/ml penicillin and streptomycin. The generation and characterization of individual cell clones from stable retroviral transduction of Panc-1 cells with a kinase-active mutant of ALK5 (T204D mutation) was described in detail in a previous publication [23]. These cells were cultured in the presence of 700 μg/ml geneticin (Life Technologies, Darmstadt, Germany). For stimulation experiments, cells were pretreated with DMSO or inhibitors (Dasatinib, SB431542) for 30–60 min before the addition of TGF-β1. The long-term treatment of Panc-1 cells with TGF-β1 was performed in standard culture medium with 2 % FCS according to a previously published protocol [32].

### Immunoblot analysis

Inhibitor-treated cells were lysed in PhosphoSafe buffer (Merck) and the protein concentrations determined with the DC protein assay (Bio-Rad, München, Germany). Equal amounts of cellular proteins were fractionated by SDS-PAGE, transferred to PVDF membrane and immunoblotted as described in detail earlier [26]. The antibodies used were: β-actin (Sigma-Aldrich, #A1978), N-cadherin (BD Transduction Lab. #610920), anti-phospho-Smad2(Ser465/467) and anti-phospho-Smad3(Ser423/425), both from Cell Signalling Technology (Frankfurt/Main, Germany), Smad2 (Epitomics, Burlingame, CA, #1736-1), Smad3 (Abcam, Cambridge, UK, #ab40854), Snail (Cell Signalling Technology, #4719), and vimentin (Sigma-Aldrich, #V6630). In some experiments, the intensities of bands were quantified by densitometry using NIH image J.

### Quantitative RT-PCR analysis

The procedure and the conditions for real-time quantitative RT-PCR (qPCR) which was performed on an I-cycler with IQ software (Bio-Rad) were published earlier [23]. Primers were generally chosen to span exon-intron boundaries. However because OCT4 is devoid of introns, amplification of its mRNA required the removal of residual genomic DNA from the isolated RNA. Therefore, RNA was prepared with the NucleoSpin® RNA isolation kit involving digestion with RNase-free DNase I (Macherey-Nagel, Düren, Germany). Sequence information for amplification primers is provided in Additional file 1 Table S1 and in Ref. [41]. All values for the genes of interest were normalised to those for β-actin and TBP, and relative gene expression was calculated by the 2^-ΔΔ^Ct method.

### Transient transfection and reporter gene assays

For reporter gene assays, Panc-1 cells were seeded in 96-well plates and cotransfected on the next day (4 h, serum-free) with LipofectAmine 2000 (Life Technologies), either one of the two TGF-β-responsive promoter-firefly luciferase reporter plasmids 3TPlux and pCAGA_(12)_-luc (kindly provided by Drs. J. Massagué and S. Dooley, respectively), and the *Renilla* luciferase encoding vector pRL-TK-luc (Promega, Heidelberg, Germany). Each well received the same total amount of DNA. Following removal of the transfection mixture, cells were allowed to recover overnight in standard growth medium and on the following day were treated with TGF-β1 [5 ng/ml] and inhibitors or vehicle (added 1 h prior to the addition of TGF-β1) for a total of 24 h. Cells were then lysed in Glo lysis buffer (Promega) and luciferase activities determined with the Dual Luciferase Assay System (Promega). In all reporter gene assays, the data for each condition were derived from six parallel wells and were corrected for transfection efficiency with *Renilla* luciferase activity.

### Real-time impedance-based measurement of cell migration

We applied the xCELLigence RTCA technology (OLS, Bremen, Germany) which represents a non-invasive and label-free approach for continuous (real-time) monitoring of cell migration and invasion on a cell culture level (for review see [42]. Considering that growth factors or their inhibitors may exert maximal effects at different time points after treatment, it is useful to monitor cell behaviour continuously and over a prolonged period of time. The precise determination of peak migratory/invasive activity of a given cell population may aid in deciphering the underlying mechanism of action of receptor kinases and their inhibitors.

Migration experiments were performed using modified 16-well plates (CIM-Plates 16, OLS ). The setup of the experimental device was described in detail earlier [27]. To begin an experiment, overnight serum-starved cells (30,000–60,000 per well) were mixed and preincubated with vehicle or dasatinib in 100 μl of culture medium containing 1 % FCS. After 30 min, TGF–β1 was added to a final concentration of 5 ng/ml and cells were seeded in the upper chambers. FCS, TGF-β1 and inhibitors had been added before to the lower chambers at the same concentrations (chemokinesis). After cell addition, CIM-Plates 16 remained at room temperature in the laminar flow hood for 30 min to allow cells to settle onto the membrane. PBS was added to the empty space surrounding the wells in order to prevent interference from evaporation. Each condition was performed in quadruplicate with a programmed signal detection every 15 min for a total of 8–24 h depending on the cell type. Data acquisition and analysis was performed with the RTCA software (version 1.2, OLS).

### Colony formation assay (CFA)

The CFAs are commonly used as survival assays to assess the cytotoxic effects of a compound on cell lines and to evaluate the efficacy of anticancer therapeutics. More recently, CFAs became an interesting method in cancer stem cell biology due to their potential to assess the capacity of cells to produce progeny. The assay relies on the assumption that only a cell which is largely undifferentiated can give rise to a countable colony. Consequently, the cell of origin must have features of a stem cell or progenitor cell. The CFA assay was carried out according to published protocols [33, 34]. Briefly, after treatment of Panc-1 cells with TGF-β ± dasatinib, cells were detached with Accutase, resuspended in culture medium and centrifuged for 5 min at 400 g. Subsequently, supernatants were discarded and cell pellets were resuspended in 1 ml of culture medium. Fourhundred cells each were seeded in three wells of a 6-well culture plate for each condition and incubated for14 days. After incubation, medium was aspirated, colonies were washed once in PBS, and fixed with 1 ml 4.5 % (*w/v*) paraformaldehyde for 15 min. Colonies were then stained with 0.1 % crystal violet for 1 h and finally washed twice in ddH_2_O and air-dried overnight. Colonies consisting of at least 50 cells were counted.

### Statistical analysis

Statistical significance was calculated from at least three independent experiments using the unpaired Student’s *t*-test. Data were considered significant at *p* < 0.05. The levels of significance are as follows: *, *p* < 0.05; **, *p* ≤ 0.01; ***, *p* ≤ 0.001.

## Additional file

Additional file 1:
**Primers used for qRT-PCR.** (DOC 51 kb)

## Abbreviations

ALK5: activin receptor-like kinase 5
CFA: colony formation assay
EMT: epithelial-to-mesenchymal transition
NSCLC: non-small cell lung carcinoma
PDAC: pancreatic ductal adenocarcinoma
SFK: SRC family kinase
TBP: TATA box binding protein
TGF-β: transforming growth factor-β
