# Supplementary material for: Dasatinib blocks transcriptional and promigratory responses to transforming growth factor-beta in pancreatic adenocarcinoma cells through inhibition of Smad signalling: implications for in vivo mode of action
Source: Mol Cancer. 2015 Nov 21;14:199. doi: 10.1186/s12943-015-0468-0 (PMC4654868; doi:10.1186/s12943-015-0468-0)
Supplement: Additional file 1: — Primers used for qRT-PCR. (DOC 51 kb) [file 12943_2015_468_MOESM1_ESM.doc]

Additional file 1: Table S1

Primers used for qRT-PCR

| Primer name | Sequence (5’→3’) |
| --- | --- |
| ABCG2-forward | gtttatccgtggtgtgtctgg |
| ABCG2-rverse | ctgagctatagaggcctggg |
| β_A_-Activin-forward | GGAGAACGGGTATGTGGAGA |
| β_A_-Activin-reverse | GGATGGTGACTTTGGTCCTG |
| BMP2-forward | AGACCTGTATCGCAGGCACT |
| BMP2-reverse | AAACTCCTCCGTGGGGATAG |
| CD90-forward | TCGCTCTCCTGCTAACAGTCT |
| CD90-reverse | CTCGTACTGGATGGGTGAACT |
| CD105-forward | CAGCTGTGGCATGCAGGTGTC |
| CD105-reverse | GTACTCACGCGTGTGCGAGTAG |
| E-cadherin-forward | TCTTCCCCGCCCTGCCAATC |
| E-cadherin-reverse | GCCTCTCTCGAGTCCCCTAG |
| GAPDH-forward | TTGCCATCAATGACCCCTTCA |
| GAPDH-reverse | CGCCCCACTTGATTTTGGA |
| MMP2-forward | CACCCTGGAGCGAGGGTAC |
| MMP2-reverse | CTGATTAGCTGTAGAGCTGAAGGC |
| MMP9-forward | CATTTCGACGATGACGAGTTGT |
| MMP9-reverse | CGGGTGTAGAGTCTCTCGC |
| N-Cadherin-forward | GTGCCATTAGCCAAGGGAATTC |
| N-Cadherin-reverse | CCTGTTCCACTCATAGGAGG |
| NCAM1-forward | AGGAGACAGAAACGAAGCCA |
| NCAM1-reverse | GGTGTTGGAAATGCTCTGGT |
| OCT4A-forward | GACAACAATGAAAATCTTCAGGAGATATG |
| OCT4A-reverse | TTCTGGCGCCGGTTACAGAACC |
| Slug-forward | ATATTCGGACCCACACATTACCT |
| Slug-reverse | GCAAATGCTCTGTTGCAGTGA |
| Snail-forward | CTGCTCCACAAGCACCAAGAGTC |
| Snail-reverse | CCAGCTGCCCTCCCTCCAC |
| TBP-forward | GCTGGCCCATAGTGATCTTT |
| TBP-reverse | CTTCACACGCCAAGAAACAG |
| UTF-1-forward | ACCAGCTGCTGACCTTGAAC |
| UTF-1-reverse | TTGAACGTACCCAAGAACGA |
| Vimentin-forward | TGGCACGTCTTGACCTTGAA |
| Vimentin-reverse | GGTCATCGTGATGCTGAGAA |
